# Phenotypic Divergence among West European Populations of Reed Bunting *Emberiza schoeniclus*: The Effects of Migratory and Foraging Behaviours

**DOI:** 10.1371/journal.pone.0063248

**Published:** 2013-05-07

**Authors:** Júlio M. Neto, Luís Gordinho, Eduardo J. Belda, Marcial Marín, Juan S. Monrós, Peter Fearon, Ross Crates

**Affiliations:** 1 Molecular Ecology and Evolution Lab, MEMEG, Department of Biology, University of Lund, Lund, Sweden; 2 CIBIO/UP-Centro de Investigação em Biodiversidade e Recursos Genéticos, Universidade do Porto, Campus Agrário de Vairão, Vairão, Portugal; 3 Instituto de Investigación para la Gestión Integrada de Zonas Costeras, Universidad Politécnica de Valencia, Gandía, Valencia, Spain; 4 Institute “Cavanilles” of Biodiversity and Evolutionary Biology, University of Valencia, Paterna, Spain; 5 Bootle, Liverpool, United Kingdom; 6 Edward Grey Institute, Department of Zoology, University of Oxford, Oxford, United Kingdom; CNRS, Université de Bourgogne, France

## Abstract

Divergent selection and local adaptation are responsible for many phenotypic differences between populations, potentially leading to speciation through the evolution of reproductive barriers. Here we evaluated the morphometric divergence among west European populations of Reed Bunting in order to determine the extent of local adaptation relative to two important selection pressures often associated with speciation in birds: migration and diet. We show that, as expected by theory, migratory *E. s. schoeniclus* had longer and more pointed wings and a slightly smaller body mass than the resident subspecies, with the exception of *E. s. lusitanica*, which despite having rounder wings was the smallest of all subspecies. Tail length, however, did not vary according to the expectation (shorter tails in migrants) probably because it is strongly correlated with wing length and might take longer to evolve. *E. s. witherbyi*, which feed on insects hiding inside reed stems during the winter, had a very thick, stubby bill. In contrast, northern populations, which feed on seeds, had thinner bills. Despite being much smaller, the southern *E. s. lusitanica* had a significantly thicker, longer bill than migratory *E. s. schoeniclus*, whereas birds from the UK population had significantly shorter, thinner bills. Geometric morphometric analyses revealed that the southern subspecies have a more convex culmen than *E. s. schoeniclus*, and *E. s. lusitanica* differs from the nominate subspecies in bill shape to a greater extent than in linear bill measurements, especially in males. Birds with a more convex culmen are thought to exert a greater strength at the bill tip, which is in agreement with their feeding technique. Overall, the three subspecies occurring in Western Europe differ in a variety of traits following the patterns predicted from their migratory and foraging behaviours, strongly suggesting that these birds have became locally adapted through natural selection.

## Introduction

Divergent selection and local adaptation are responsible for many phenotypic differences found across populations, and may lead to the evolution of reproductive barriers and speciation [1], [2]. Local adaptations are expected to constrain gene flow among populations, as hybrids would be maladapted relative to their parents [1]. In addition, the speciation process is greatly facilitated, even in the presence of gene flow, when the traits subject to divergent selection are also involved in mate choice (often called ‘magic traits’ [3]). In order to understand the speciation process, it is important to determine how ecology and genetics interact to cause the evolution of the first reproductive barriers, before they are confounded by further barriers and differences evolving subsequently among populations/species [4]. The characterization of diverging phenotypes and the identification of relevant evolutionary forces acting on those phenotypes are crucial first steps to study the causes of speciation [5].

In birds, two of the most significant selection pressures associated with the evolution of reproductive barriers are migratory and foraging behaviours. For instance, reproductive isolation seems to be evolving as a consequence of a new migratory direction in Blackcaps (*Sylvia atricapilla*; [6]), and migratory behaviour has been suggested to be an important factor promoting speciation [7]–[11]. On the other hand, foraging ecology has been associated with divergent selection and speciation, particularly in seed-eating species such as Darwin’s finches, *Nesospiza* buntings and crossbills [12]–[14]. Other organisms have also evolved in foraging behaviour leading to speciation, such as the benthic and limnetic threespine sticklebacks [15]; and niche divergence has been shown to promote reproductive isolation in a large variety of taxa [16].

The Reed Bunting (*Emberiza schoeniclus*) is the most variable species within the large *Emberizidae* family, having numerous subspecies described on the basis of bill size, body size and plumage colour [17]–[20]. The variation in phenotype is complex and to a large extent clinal [19], [20]. Birds with a thick bill occur in the southern part of the distribution, where the thickness of the bill (as well as body size) increases towards the east, whereas thin-billed birds occur further north. In addition, western individuals are the darkest in plumage, becoming increasingly light in colour towards the east [17], [19], [20]. Southern populations are resident, but further north partial, short- and medium-distance migration occurs in various directions, with thin-billed subspecies often co-occurring with thick-billed birds during the winter [21]. During spring and summer, Reed Buntings feed mostly on insects, but during the winter, thick-billed birds seem to feed on insects lying dormant inside the reed (*Phragmites australis*) stems; whereas the thin-billed birds feed almost exclusively on small seeds (Shtegman 1948 cited by [21], personal observations) [22]–[24].

Individual variation and the existence of intermediate populations led to some instability in Reed Bunting’s taxonomy, with the number of recognized subspecies varying from 15 to over 20 [17]–[20], [25], [26]. One of the subspecies for which little data exist and has not been recognized by most authors before Byers et al. [20] is *E. s. lusitanica* (hereafter *lusitanica*; first described by Steinbacher [27]), which resides in the northwest part of the Iberian Peninsula (see Figure S1, [28]). It was lumped with *E. s. witherbyi* (hereafter *witherbyi*) pending further study by Vaurie [17] and by Cramp & Perrins [19], though they admitted that it should probably belong to the thin-billed group, close to *E. s. schoeniclus* (hereafter *schoeniclus*), as was later described by Byers et al. [20].

Previous studies addressing phenotypic divergence amongst Reed Bunting subspecies generally analysed very few individuals of each population and no statistical comparisons were made (but see [29], [30]). Genetic studies, however, have shown that the neutral genetic divergence between the Italian subspecies *E. s. intermedia* (thick-billed) and the central-European *schoeniclus* (thin-billed) is slight but significant [31]. This was confirmed by a recent analysis of mitochondrial DNA (ND2 gene) describing three partially overlapping closely-related lineages in Asia [32], and by our own analysis of mtDNA (control region) and microsatellites of Iberian and central European subspecies [33]. Song discrimination between different subspecies is also slight [34], but the bill size differences between *E. s. intermedia* and *schoeniclus* are correlated with diet suggesting local adaptation [22]. Furthermore, there seems to be no interbreeding between thick-billed and thin-billed subspecies in contact zones [31]. Therefore, this species seems to be at an early stage of speciation, with populations/subspecies still retaining ancient polymorphisms, but showing significant genetic, morphological and behavioural divergence. Bill and body size are especially interesting, as these traits are likely to influence song characteristics involved in mate choice [35] (Gordinho et al. in prep), potentially acting as magic traits of (ecological) speciation [3]. It is particularly interesting to study organisms at this stage of evolution, when the actual ecological and genetic mechanisms of speciation can be witnessed.

In this study, we evaluated the morphometric divergence among west European populations including two resident southern subspecies from the Iberian Peninsula, *witherbyi* and *lusitanica*, as well as migratory and resident populations of *schoeniclus*. Our purpose was to determine the extent of local adaptation, evaluating the effects of migratory behaviour and diet/feeding technique, and to describe for the first time the morphometrics of *lusitanica*. In particular, we tested the expectations that migratory birds should have longer and more pointed wings, shorter tails and lower body mass than residents [36], [37]. In addition, we evaluated to which extent *lusitanica* differed from *witherbyi* and *schoeniclus* in terms of bill size and shape. As a southern resident subspecies, *lusitanica* is expected to feed on insects lying inside reed stems during winter (Neto et al. in prep), thus being close to *witherbyi* in foraging-related traits, even though recent authors include it in the small-billed group [20]. Morphological characters such as the ones analysed here are generally highly heritable [38], [39], and given that the genetic divergence is very small [33], the morphological differences among populations are likely to be meaningful (adaptive), especially if the predictions are confirmed, showing that the individuals “fit” their environments. It is especially useful to study local adaptation in the west European populations of Reed Bunting because *schoeniclus* includes both resident and migratory populations, and Iberia is inhabited by two resident populations/subspecies that differ markedly in size and bill characters thereby allowing to separate the effects of migration and foraging. With its intermediate characteristics, *lusitanica* is of considerable interest because it allows us to evaluate the level of reproductive isolation between the two bill-size groups.

## Materials and Methods

### Fieldwork

**Figure 1:**
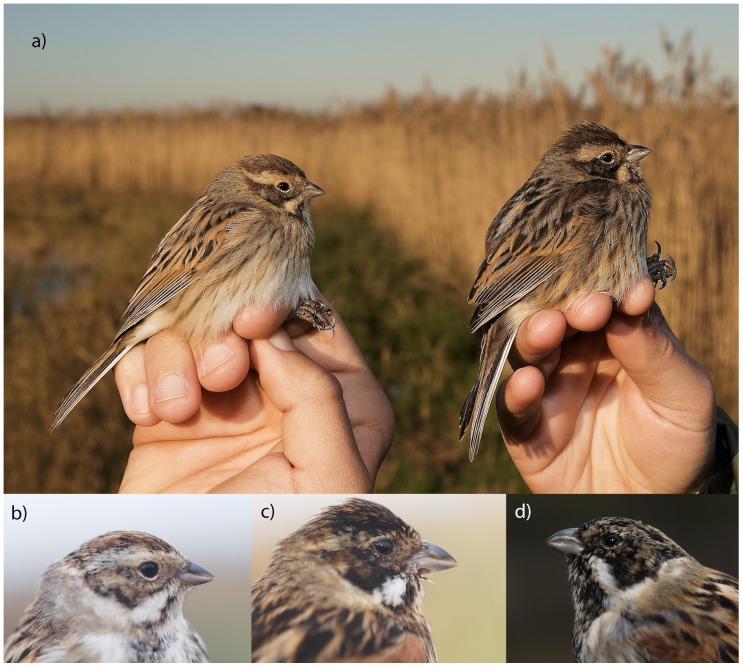
Examples depicting plumage and bill shape differences among Reed Bunting subspecies. a) first-year females *E. s. schoeniclus* (left) and *E. s. lusitanica* (right); b) first-year male *E. s. schoeniclus*; c) first-year male *E. s. lusitanica* and d) first-year male *E. s. witherbyi*, captured at Salreu, Estarreja, Portugal, except the latter, which was captured at Lagunas de Villafranca, Toledo, Spain. All pictures were taken by JMN.

Biometric data of Reed Buntings were obtained from several populations (Figure S1): (1) the resident *lusitanica* was measured at Salreu marshlands, Portugal, from 2008 to 2011 (n = 201); (2) the resident *witherbyi*, measured at several sites in Spain from 2002 to 2012 (n = 76); (3) wintering *schoeniclus* measured at Salreu marshlands from 2008 to 2011 (n = 94); (4) the resident *schoeniclus* from the United Kingdom, sampled in the Liverpool and Oxford regions in autumn 2011 (n = 47); and (5) Scandinavian migrants (*schoeniclus*) sampled at lake Krankesjön, Skåne, Sweden, just prior to autumn migration in 2011 (n = 22). The two subspecies that occur in Salreu were distinguished on the basis of date and plumage traits, with *lusitanica* being obviously darker in the head, upper parts and flanks, and having also darker and more intensely-coloured wing coverts than the wintering *schoeniclus* (Figure 1, see also [20], [26]). Judging from the many local and foreign retraps, the experience gathered during the last few years allowed us to classify each bird to subspecies with 100% certainty, although there are no quantitative data on plumage traits. Spanish birds of the subspecies *witherbyi* were distinguished from the wintering *shoeniclus* on the basis of date, plumage and genetics [30].

Birds were captured with mist-nets, marked with a metal ring issued by the ringing centre of the country where ringing took place, and the age and sex were determined using published criteria [40], [41]. The wing (maximum chord) and tail lengths were measured with a ruler to the nearest 0.5 mm, tarsus and bill (to skull) lengths, bill depth and bill width (at the nostrils) were measured with callipers to the nearest 0.1 mm, weight was measured either with a Pesola spring balance or a digital balance to the nearest 0.1 g and the subcutaneous fat reserves were recorded following Kaiser [42]. In addition, the length of each primary was measured as described by Jenni & Winkler [43] in birds with fresh feathers in autumn and winter. The sample size for each individual measurement is variable, as it was not possible to measure all traits in all birds.

The Portuguese (*lusitanica* and wintering *schoeniclus*) and Swedish Reed Buntings were measured by JMN, whereas Spanish birds were measured by JMN, MM, JSM, EJB and others, and the birds from the U.K. were measured by PF and RC. Differences in measuring technique between the ringers (especially wing and bill lengths, which are more difficult to measure) could potentially be a problem for population comparisons because they will result in significant differences given enough sample size. However, JMN and PF have been ringing together for many years and their measurements were calibrated and are comparable (the same was done at a later stage between JMN, MM and JSM). In addition, although preliminary analysis showed that many statistical comparisons between *schoeniclus* wintering in Portugal (measured by JMN) and in Spain (by several Spanish ringers) are significant (despite these birds probably having the same origin and biometrics), the actual differences in the means are very small relative to the differences that we found among the populations/subspecies. For instance, the difference in wing length (when controlling for age and sex) between Portuguese and Spanish *schoeniclus* was only 1.37 mm (F_[1;744]_ = 29.8; P<0.001), whereas the difference in tail length was 0.84 mm (F_[1;737]_ = 2.76; P = 0.097), tarsus 0.36 mm (F_[1;741]_ = 20.0; P<0.001) and bill depth 0.18 mm (F_[1;683]_ = 34.1; P<0.001; see also Gosler et al. [44] for a general inter-observer comparisons of measurements of the same individual birds). Hence, the phenotypic divergence found between populations (see Table 1) is real and not caused by inter-observer differences. Furthermore, analyses restricted to birds measured by JMN produced qualitatively similar results (although the UK population was not included), and so we provide the results obtained from the full dataset.

**Table 1:** Unstardardized parameters and t-tests of the General Linear Models evaluating the effects of age, sex and subspecies/population on the various biometrics.

	Age	Sex	Population
**Wing**	−1.454±0.211***	−5.652±0.210***	(lus) −1.545±0.370***
			(sch) 3.543±0.377***
			(UK) 2.351±0.436***
**Tail**	−0.898±0.252**	−3.619±0.251***	(lus) −3.861±0.464***
			(sch) −0.524±0.471ns
			(UK) −2.087±0.534***
**Tarsus**	0.017±0.093ns	−0.650±0.091***	(lus) −0.959±0.153***
			(sch) −0.603±0.157**
			(UK) 0.242±0.186ns
**Bill length**	−0.009±0.054ns	−0.390±0.053***	(lus) 0.511±0.089***
			(sch) 0.278±0.091**
			(UK) −0.275±0.117**
**Bill depth**	**−**0.052±0.027#	−0.254±0.027***	(lus) −0.911±0.045***
			(sch) −1.094±0.047***
			(UK) −1.411±0.055***
**Bill width**	**−**0.039±0.040ns	−0.135±0.039***	(lus) −1.305±0.066***
			(sch) −1.314±0.068***
			(UK) −1.728±0.086***
**Bill shape index**	**−**0.027±0.014#	0.035±0.014*	(lus) 0.437±0.023***
			(sch) 0.479±0.023***
			(UK) 0.530±0.030***
**Body mass**	**−**0.155±0.128ns	−2.238±0.127***	(lus) −1.917±0.232***
			(sch) −0.625±0.239**
			(UK) 0.056±0.276ns
**Tail/Wing**	**−**0.004±0.003ns	0.015±0.003***	(lus) −0.033±0.005***
			(sch) −0.045±0.005***
			(UK) −0.051±0.006***
**PC1_WING_**	0.205±0.179ns	0.282±0.180ns	(lus) −1.707±0.416**
			(sch) −2.126±0.396***
			(UK) −1.379±0.394***
**PC2_WING_**	0.598±0.176***	0.466±0.176**	(lus) −1.049±0.408**
			(sch) **−**0.117±0.38ns
			(UK) 0.259±0.386ns
**PC_BILL_**	0.008±0.057ns	−0.469±0.056***	(lus) −1.936±0.093***
			(sch) −2.207±0.095***
			(UK) −3.047±0.132***
**PC_SIZE_**	0.293±0.067***	−1.394±0.067***	(lus) −1.061±0.124**
			(sch) 0.058±0.126ns
			(UK) **−**0.058±0.151ns
**RW1**	**−**0.004±0.005ns	−0.026±0.005***	(lus) −0.112±0.013***
			(sch) −0.151±0.013***
			(UK) −0.163±0.014***

# – P = 0.059; *** – P<0.001; ** – P<0.01; * – P<0.05; ns – non-significant.

Fat and muscle scores were included as covariates in the model analysing body mass. The parameters represent the difference relative to adults, males and *E. s. witherbyi*. Models with significant interactions are presented in the main text.

### Geometric Morphometrics of the Bill

A photograph of the bill in profile was taken from 208 individuals of all populations/subspecies, and subjected to geometric morphometric analysis, a powerful method with few *a priori* assumptions to explicitly define shape [45]–[48]. This method has recently been applied to a growing number of animal groups, including in a few bird studies that compare bill shapes [49]–[51]. Prior to analysis, photographs were edited in Adobe Photoshop CS4 (for details see Protocol S1), and then all geometric morphometric analyses were conducted in software of the tps series [52]. A tps file was built from images using tpsUtil [53], [54] and used in tpsDig [55], where seven landmarks and eight semi-landmarks were digitized following Foster et al. [49]. The semi-landmarks were placed by reference to a standardized grid superimposed onto each image (cf. Figure S2 and Protocol S2). Files containing links (between landmarks) and sliders (for each semi-landmark) were built in tpsUtil and an image list was obtained. Using the tpsSmall software [56], we confirmed that shape variation between the specimens was sufficiently small and therefore the distribution of points in the shape space can be represented satisfactorily by their distribution in the tangent space. We then applied a Generalized orthogonal least-squares Procrustes Analysis (GPA) [57], [58] using tpsRelw [59], in order to standardize the size and to translate and rotate the configurations of landmark coordinates, therefore obtaining a consensus configuration. We computed partial and relative warps and extracted relative warp scores with a α = 0, using the tpsRelw software [59]. tpsRelw output files were saved in NTS format, converted to csv using tpsUtil, and merged with the image list in Microsoft Excel. Because of logistical constraints that prevented inclusion of a standardized scale in each image, allometry was evaluated by reference to a Principal Component based on univariate measurements [49] (see below).

### Statistical Analysis

As the variables were approximately normally distributed and there were no obvious deviations from model assumptions judging from the variance comparisons, covariance structure and residuals, General Linear Models (GLM) were used to determine and evaluate the effects of age, sex and population/subspecies on each trait. Two-way interactions were also tested and kept in the final model if significant. The basic biometrics (wing, P8, tail, tarsus, bill length, bill depth and bill width) were included in stepwise (forward) discriminant analyses (using default parameters, i.e., a variable was entered in the model if it improved significantly the significance of Wald’s test, having an F >3.84, and dropped if F <2.71) in order to determine to which extent birds of different subspecies and populations were correctly classified and by which variables.

The size of the feeding apparatus (bill length, depth and width) was reduced to one variable using principal component analysis (PC_BIlL_, Table S1), which represents overall bill size and explains 60.1% of the variance. A bill shape index was calculated by dividing bill length by bill depth. Tail to wing ratio was also calculated for each bird by dividing these variables. The primary lengths were first corrected for body size isometrically following Lleonart et al. [60] and using a standard wing length of 78 mm. Subsequently, adjusted primary lengths were reduced to two variables (representing wing shape) using principal component analysis (PC1_WING_ and PC2_WING_), which explained 46.6% and 21.0% of the variance, respectively (Table S2). PC1_WING_ represents (the inverse of) wing convexity, as it is strongly correlated with the length of the inner primaries, but not with the outer primaries (Table S2); whereas PC2_WING_ reflects wing pointedness because it is strongly correlated with the longest primaries (Table S2, see also [8], [29]). Overall body size, estimated as the first principal component of an analysis including wing, tail, tarsus and bill lengths (PC_SIZE_, 51.7% of variance explained, Table S3), was included as a covariate in some analyses in order to control for allometric differences. Whenever one of the four variables contributing to PC_SIZE_ was the dependent variable in the statistical model, body mass (and fat score) were used as covariates to control for allometry. Statistical analyses were undertaken in SPSS 20.0 [61], and results are presented as mean ± SE (n).

### Ethical Treatment of Animals

The capture and ringing of birds was conducted under the licenses required by the corresponding national authorities, following standard protocols and releasing the birds unharmed on site. Permits were given by the following institutions: Daimiel National Park, Marjal Pego-Oliva Natural Park, S’Albufera de Mallorca Natural Park, Conselleria de Medi Ambient, Aigua, Urbanisme i Habitatge, Generalitat Valenciana (440066); Consejería de Medio Ambiente y Desarrollo Rural de Castilla La Mancha; Direcció General de Medi Natural, Educació Ambiental i Canvi Climàtic, Conselleria d'Agricultura, Medi Ambient i Territori, Govern de les Illes Balears (13123/2012); Consejería de Medio Ambiente de la Junta de Andalucía (6305); Ringmärkningscentralen, Naturhistoriska Riskmuseet; CEMPA, Instituto de Conservação da Natureza e Florestas (99/2011, 112/2012); British Trust for Ornithology (RC = 5435, AF5394).

## Results

### General Morphological Differences

Swedish birds were statistically indistinguishable in all traits (GLM, all P>0.1) to the *schoeniclus* wintering at Salreu, Portugal, which, according to ringing controls, originate from northern France, Sweden, Germany, Poland and Czech Republic (Neto et al., in preparation). Therefore, these two populations were lumped in all subsequent analyses. Otherwise, biometrics differed markedly among the studied populations/subspecies (Tables 1, Table S4). Age significantly influenced the length of feathers (wing and tail) and consequently body size (PC_SIZE_), with adults being larger than first-year birds. Also, with the exception of wing shape (PC_WING_), all measurements differed between the sexes, with females being significantly smaller than males, but having higher values of bill shape index (bill length/bill depth) and tail/wing ratio. Hence, these factors had to be taken into account for population comparisons.

**Figure 2:**
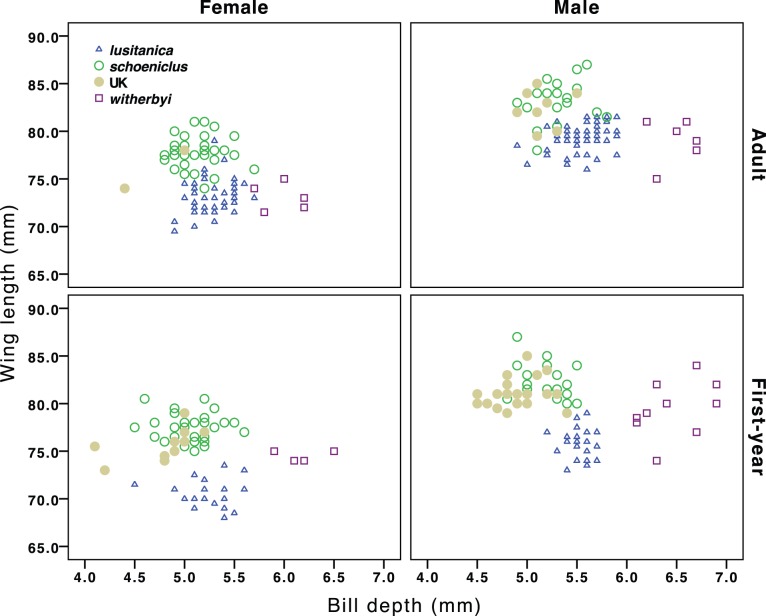
Scatterplot of bill depth and wing length for each age, sex and subspecies/population. *E. s. schoeniclus* includes birds trapped in Portugal during winter as well as those measured in Sweden.

Stepwise discriminant analysis indicated that 100% of male (Wilk’s lambda = 0. 142, χ^2^ [_4_] = 161.83, P<0.001) and 97.9% of female (Wilk’s lambda = 0.192, χ^2^ [_4_] = 108.89, P<0.001) *witherbyi* can be correctly distinguished from *lusitanica* (and from the other populations studied here) on the basis of bill depth, bill width, bill length and tarsus length (but note that bill depth alone was enough to correctly classify 100% of male and 98% of female *witherbyi* from *lusitanica*; see also [30]). Wing length, bill depth and bill width allowed the correct classification of 94.8% of male (Wilk’s lambda = 0.328, χ^2^ [_3_] = 109.70, P<0.001) and bill and wing lengths 92.6% of female (Wilk’s lambda = 0.321, χ^2^[_2_] = 142.09, P<0.001) *lusitanica* and *schoeniclus* (see Figure 2). On the other hand, discriminant functions of the two populations of *schoeniclus* (migratory and UK residents) were able to correctly classify 88.3% of male (Wilk’s lambda = 0.542, χ^2^ [_3_] = 30.91, P<0.001) and 71.4% of female (Wilk’s lambda = 0.943, χ^2^ [_1_] = 4.043, P = 0.044) on the basis of bill width (both sexes), bill depth and tarsus length (the latter two for males only).

### Adaptations to Migration

Body mass (with fat and muscle scores as covariates) was similar between *witherbyi* and *schoeniclus* resident in the UK, but was slightly, but significantly, smaller in migrant *schoeniclus* and even smaller in *lusitanica*. Body size (PC_SIZE_), however, was similar across populations except for *lusitanica*, which was significantly smaller than the other subspecies (Table 1). The discrepancy in the comparisons of body mass and body size across populations can be explained by migrant *schoeniclus* having the longest wings (Table 1, Table S4), which was the most important factor loading for PC_SIZE_ (Table S3). Although *lusitanica* appeared equally small in mass and size (PC_SIZE_) relative to the other subspecies, it actually had the longest bill, but was smaller in all other body measurements (wing, tail, tarsus; Table 1).

**Figure 3:**
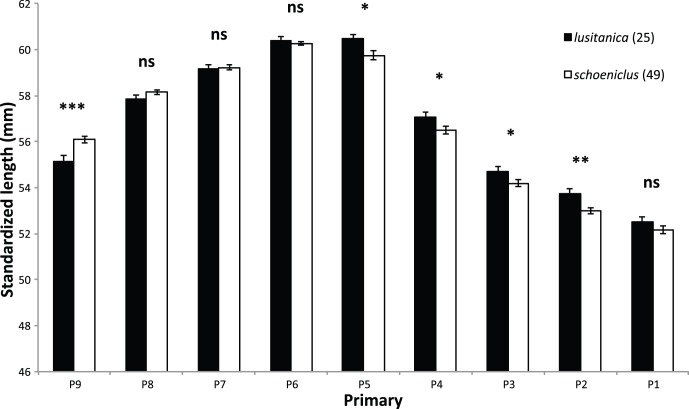
Isometrically-adjusted primary lengths of the resident *E.* **s. lusitanica**** **and the migratory** ***E. s. schoeniclus*** **wintering in Portugal and from Sweden.** **** Sample size is indicated between parentheses. T-tests indicate that primaries 9, 5, 4, 3, and 2 are significantly different between the subspecies (ns – non-significant; * – P<0.05; ** – P<0.01; *** – P<0.001).

As predicted by theory, migratory populations of *schoeniclus* had the longest wings, followed by resident *schoeniclus* from the UK, *witherbyi* and *lusitanica*, which had almost no overlap in wing length with the other populations (Table 1, Figure 2, Table S4). Wing convexity (PC1_WING_) also varied significantly across populations, with migratory *schoeniclus* having the most negative values (i.e. more convex wings), followed by *lusitanica*, resident *schoeniclus* from the UK and *witherbyi* (Table 1; see also [29]). On the other hand, *lusitanica* had significantly less pointed (PC2_WING_) wings than the remaining populations, which were otherwise similar (Table 1). Differences in wing shape are better illustrated between *lusitanica* and the migratory *schoeniclus*, as both have a large sample size and were measured by the same person (JMN), allowing for detailed comparisons between the primaries (Figure 3). As predicted by theory, migratory birds had significantly longer outer primaries and shorter inner primaries, and a tendency to have P6 longer than P5, whereas in *lusitanica* P5 seems slightly longer on average than P6 (Figure 3). The inclusion of body size (PC_SIZE_) as a covariate in the statistical model does not affect the comparison of wing shape (PC1_WING_ and PC2_WING_) between populations (GLM: PC1_WING_: PC_SIZE_: F_[1;96]_ = 0.49, P = 0.486; Age: F_[1;96]_ = 0.91, P = 0.342; Sex: F_[1;96]_ = 1.56, P = 0.215; Population: F_[3;96]_ = 9.92, P<0.001; PC2_WING_: PC_SIZE_: F_[1;96]_ = 0.49, P = 0.486; Age: F_[1;96]_ = 9.77, P = 0.002; Sex: F_[1;96]_ = 0.01, P = 0.919; Population: F_[3;96]_ = 6.38, P = 0.001), and so the difference is not caused by allometry.

Although the tail of the migratory populations of *schoeniclus* was significantly longer than that of the resident *schoeniclus* from the UK and of *lusitanica* (but not significantly different from *witherbyi*), the tail/wing ratio was significantly smaller in migratory *schoeniclus* than that of other subspecies except the resident UK population (GLM with *schoeniclus* and males as reference and B representing the unstandardized coefficients/parameters of the fitted model: Sex: B = 0.014±0.03, P<0.001; Population: (lus) B = 0.010±0.003, P = 0.001, (UK) B = **−**0.008±0.004, P = 0.066, (wit) B = 0.041±0.005 P<0.001). However, if body mass (rather than PC_SIZE_, which depends on tail length) is used as a covariate to adjust for differences in body size, the tail length of migratory *schoeniclus* and *witherbyi* are not significantly different (B = 0.255±0.494 mm, P = 0.605), whereas the tail of *schoeniclus* from the UK are significantly shorter (B = **−**1.966±0.443 mm, P<0.001) and even shorter in *lusitanica* (B = **−**2.705±0.317 mm, P<0.001).

### Adaptations to Foraging

Although there is a large overlap in measurements, all bill traits differed significantly between *lusitanica* and *schoeniclus*, particularly bill depth and width, the former being 2.6–3.9% (females–males) larger in *lusitanica* (Table 1, Figure 2). This is particularly remarkable given that *schoeniclus* is 7.4–8.3% heavier and have 6.1–4.9% longer wings than *lusitanica* (Table S4). As described above, there was virtually no overlap in bill depth between the thick-billed *witherbyi* and the remaining subspecies, with *witherbyi* having a bill 14.3–17.3% deeper than *lusitanica*, but being only 8.0–7.8% heavier (Figure 2, see also [30]). On the other hand, resident *schoeniclus* from the UK had significantly shorter (3.2–5.3%) and less deep (7.3–8.6%) bills than the migratory *schoeniclus* (Table 1, Figure 2). In contrast to the measurements of the flight apparatus, there were significant interactions (not shown in Table 1) between population and sex in bill length (F_[3;317]_ = 2.97, P = 0.032) and bill depth (F_[3;323]_ = 3.98, P = 0.008), which result from the fact that males differ more between populations than females in these traits (see Figure 2 and above). The inclusion of PC_SIZE_ as a covariate in the model comparing bill depth between populations still resulted in highly significant differences (GLM: PC_SIZE_: F_[1;281]_ = 23.2, P<0.001; Sex: F_[1;281]_ = 2.3, P = 0.130; Population: F_[3;281]_ = 190.2, P<0.001; Sex vs. Population: F_[3;281]_ = 3.9, P = 0.009). Hence, allometry does not explain the patterns found, particularly between *witherbyi* and *lusitanica*, which vary in size and bill depth in the same direction. Bill width largely follows the variation described for bill depth, as does the overall bill size (PC_BILL_), whereas the bill shape index varied in the opposite direction with *witherbyi* having the deepest bill in relation to its length, followed by *lusitanica*, migratory *schoeniclus* and the resident *schoeniclus* from the UK (Table 1).

**Figure 4:**
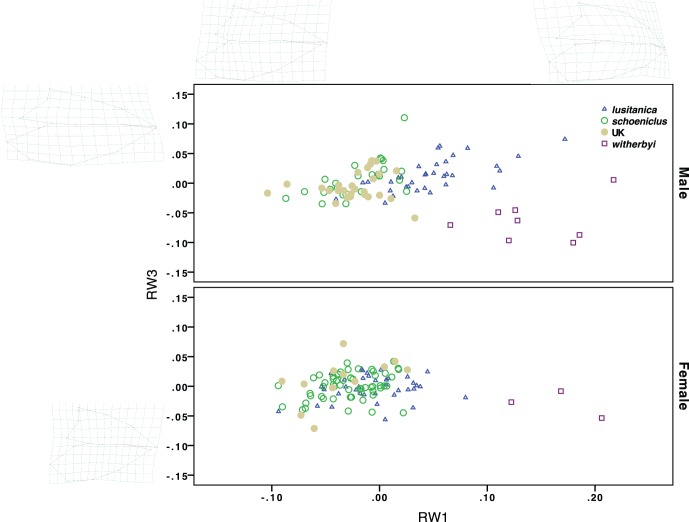
Bill shape in relation to population and sex, as measured by the two most important axis of variation for population discrimination (RW1 and RW3) derived from geometric morphometric analysis.

Geometric morphometrics of the bill in profile revealed significant differences for the first nine axis (RW1-9) of variation between the populations/subspecies (for RW1 see Table 1). The first axis (RW1), which is the most important for population discrimination, represents variation in the curvature of the culmen, with *witherbyi* having the highest values, followed by *lusitanica*, migratory *schoeniclus* and then by resident *schoeniclus* from the UK (Table 1, Figure 4). As with the linear measurements, the interaction between sex and population is highly significant (F_[3;190]_ = 5.78, P = 0.001) because females do not differ as much between populations as males (see Figure 4). When body size (and birds of unknown age, since age is not significant, see Table 1) is included in the statistical model, the comparisons among populations and the interaction with sex, remain highly significant (GLM: PC_SIZE_: F_[1;174]_ = 0.33, P = 0.569; Sex: F_[1;174]_ = 1.86, P = 0.174; Population: F_[3;174]_ = 32.11, P<0.001; Sex vs. Population: F_[3;174]_ = 4.754, P = 0.003), and so differences in bill shape cannot be explained by allometry. RW3, the second most important bill shape variable to discriminate the populations (representing variation from short, stubby to long, shallow bills, see Figure 4), produces similar results to RW1 (GLM: PC_SIZE_: F_[1;174]_ = 0.23, P = 0.629; Sex: F_[1;174]_ = 1.82, P = 0.179; Population: F_[3;174]_ = 5.93, P = 0.001; Sex vs. Population: F_[3;174]_ = 3.03, P = 0.031). The difference in RW1 between *lusitanica* and migratory *schoeniclus* is greater than for any linear measurement of the bill, especially in males (Figure 4). Indeed, discriminant analyses (using RW1-5) between these two populations resulted in 95.1% of the males and 75.5% of females being correctly classified to their original population; whereas linear measurements of the bill (length, depth, width and bill shape index) resulted in 80.2% of the males and 74.7% of the females being correctly classified.

## Discussion

In this study, we described the phenotypic divergence amongst Reed Bunting populations likely to be relevant for the seemingly on-going speciation process in this species. We chose to analyse traits for which clear predictions of the direction of evolution could be made relative to two selection pressures that are known to influence speciation in birds: migration and diet. In particular, we showed that, according to predictions, migratory *schoeniclus* had longer and more convex wings than the resident Iberian subspecies (see also [29]), and similar patterns have been found in other bird species [37], [39], [62], [63]. The migratory *schoeniclus* also had slightly smaller body mass than the other populations, as predicted by theory, except for *lusitanica*, which is much smaller than the other subspecies. These variations/adaptations seemed to have occurred despite northern Reed Buntings being short to medium distance migrants, rather than long-distance migrants [21], and so the selection pressure for high aspect-ratio wings might not be as high as in other species that have been studied (e.g. [37]). Comparisons of migratory and resident populations of the nominate subspecies reveal slight differences in wing shape, which is rounder (less convex) in the resident population (UK) than in migratory *schoeniclus*. Also, birds from the UK were heavier than the migrants, but in contrast to expectations had relatively short tails. A recent common ancestry, large gene flow and the occurrence of short-distance seasonal movements in UK birds (particularly in some years when snow cover might prevent them to have access to seeds; [21], [64]) might explain the small differences found. Tail length, however, did not vary according to the expectation of shorter tail in migratory birds, and tail/wing ratio seemed to reflect mostly the longer wings of migrants (see also [37]). This may be a consequence of tail and wing lengths being strongly correlated both phenotypically and genetically in birds, and for this reason it is possible that tail length takes longer to evolve and may even act as a morphological constrain to adaptation in wing lengths [65].

We also show that the southern subspecies, which have been observed feeding on dormant insects lying inside reed stems during winter, have thicker bills (which they use to open the reed stems; pers. observations, [21]). In contrast, northern populations, which switch their diets to seeds during the winter [23] (although they can also feed on insects opportunistically by gleaning [24]; pers. observations), have much shorter and especially thinner bills (see also [30]). Particularly interesting is the small, resident, Iberian subspecies *lusitanica*, for which we present for the first time statistical comparisons with other populations. This subspecies has a disproportionally long and thick bill for its small body size, having a significantly larger bill than the large-bodied nominate subspecies, but smaller/thinner than all *witherbyi*. In contrast, birds from the UK have smaller and thinner bills than those of migratory *schoeniclus*, which could be associated with a diet composed of smaller seeds (although this has so far not been studied in any detail).

In addition to the linear measurements, our geometric morphometric analyses revealed important differences in bill shape, particularly in culmen curvature. The resident *witherbyi* remains especially distinct regarding bill shape from the remaining populations; but in contrast to the linear measurements, there is some overlap in culmen curvature (RW1) between *lusitanica* and *witherbyi* males (Figure 4). On the other hand, *lusitanica* differs from the nominate subspecies in bill shape to a greater extent than when only the linear bill measurements are used, especially in males, although there is still overlap between these subspecies (Figure 4). This is most likely associated with differences in diet (Neto et al., in prep.) because birds with a more convex culmen are able to exert a greater strength at the bill tip, which is probably very useful to open the reed stems, whereas seed-eating birds tend to crack the seeds at the base of the bill [49]. Given these results, and despite the overlap in bill traits with *schoeniclus* (especially in females), *lusitanica* appears to share morphological traits with the thick-billed subspecies (as expected by their ecology and distribution), but it is still quite distinctive from both groups due to its much smaller size and dark plumage (in addition to the feeding apparatus).

One interesting morphological difference clearly shown by our analyses is the sexual dimorphism in bill size and shape, which is consistent across subspecies. Sexual differences in bill size and shape do not result from the overall small body size of females, as sex remains significant when body size is taken into account in the statistical models. Females have shorter, thinner bills and a less convex culmen than males and, independently of its origin (sexual selection or intra-specific competition), these differences are probably associated with ecological differences that have hitherto not been studied. It is possible that females prefer smaller seeds in northern populations or search for insects in thinner reeds in southern populations, but more radical foraging niche differences may occur between the sexes. Interestingly, bill size and shape diverged more between populations in males than in females, which could suggest that in addition to ecology, sexual selection could have also played a role in population divergence. Our results are comparable to those described for tidal-marsh (North American) sparrows, for which intraspecific competition for food (and/or possibly male-male competition for territories/females) was considered the most likely cause for the greater increase in male than female bill size associated with the colonization of marshes by a variety of emberizid species [66]. As shown theoretically and empirically (in threespine stickebacks), both sexual dimorphism and speciation can co-occur as long as the effects of loci underlying sexually dimorphic traits are orthogonal to those underlying sexually selected traits [67]. The role of sexual selection and competition in producing the sexual differences found in Reed Buntings deserve further research.

Another interesting morphological difference that we described is the much smaller size of *lusitanica* relative to the remaining subspecies, for which we have no obvious adaptive explanation. This subspecies lives in close proximity to the large and thick-billed *witherbyi*, but uses mostly coastal reedbeds located in the Atlantic influenced (wet, mild) geographical region, whereas the latter occurs mostly in inland (occasionally coastal) reedbeds in the Mediterranean influenced (dry, hot or continental) region. Both the small size and dark plumage of *lusitanica* could perhaps be explained by adaptations to the mild, wet climate where they occur (following Gloger’s rule); whereas its thinner bill (in comparison with *witherbyi*) could be related to their occurrence in brackish sites, where the reeds tend to be shorter and thinner, although this is not sufficiently studied. As *witherbyi* have a thicker bill than *lusitanica*, even when controlling for body size, and the foraging ecology seems to be similar (Neto et al. in preparation), it is possible that bill size between these subspecies has evolved to dissipate heat in the warmer eastern Iberian sites. In fact, summer temperatures might be responsible for the clinal variation of increasing bill size towards the east among thick-billed subspecies of Reed Buntings. This has recently been shown to occur in several North American emberizids [68]–[70]. The relative roles of diet and temperature on the evolution of bill size should be further studied in Reed Buntings, especially among subspecies with similar diets.

In previous studies, we have shown that the genetic divergence among the Reed Bunting subspecies is very small, but significant, with *G*_ST_ (microsatellites) ranging from 0.03 to 0.04 and *Φ*_ST_ (mtDNA) from 0.04–0.05 between *schoeniclus* and each Iberian subspecies; and 0.04 (microsatellites) and 0.14 (mtDNA) between the two Iberian subspecies [33]. In addition, the shallow mtDNA phylogeny indicates that these subspecies diverged very recently, after the last glacial maxima [33]. Therefore, and given that the morphological traits studied here generally have high heritabilities [38], [39] and showed limited plasticity in a common garden experiment with a North American emberizid [71], differences among populations found in this study probably evolved very rapidly through natural selection. However, genetic drift, especially in the threatened Iberian subspecies, cannot be excluded as a potential explanation for some of the morphological differences that were found, nor does (adaptive) plasticity. Detailed comparisons between genetic and phenotypic divergence are clearly needed (for which additional genetic markers need to be used relative to those already available for this system [33]), as well as common garden experiments, in order to confirm whether these traits are indeed under selection or locally adapted [71], [72].

In conclusion, our morphometric analyses clearly show that the three subspecies of Reed Buntings occurring in Western Europe differ in a variety of traits in the direction predicted by their migratory and foraging behaviours, strongly suggesting that these birds became locally adapted through natural selection. Whether these traits contribute to reproductive isolation is currently being investigated in this interesting study system (Gordinho et al, in preparation). This study contributes to improve upon the limited knowledge on speciation phenotypes that is available for a variety of organisms [5].

## Supporting Information

Figure S1 **Approximate breeding distributions of Reed Bunting subspecies occurring in Europe.** (based on [17]–[20]). Sampling sites are indicated with a red star.
(EPS)

Figure S2 **Location of the seven landmarks and eight semi-landmarks (calculated from the landmarks) used in geometric morphometric analyses.**
(TIF)

Table S1 **Principal component analysis of bill size measurements, used to extract PC_BILL_.**
(DOC)

Table S2 **Principal component analysis of the isometrically-adjusted primary lengths, used to extract PC1_WING_ and PC2_WING_, which represent wing convexity and wing pointedness, respectively.**
(DOC)

Table S3 **Principal component analysis of body size measurements, used to extract PC_SIZE_.**
(DOC)

Table S4 **Descriptive statistics of morphological traits for each population, sex and age class. Individuals captured in Sweden did not differ from individuals of the nominate subspecies wintering in Portugal, and so they were lumped.**
(DOC)

Protocol S1 **Photograph editing in Photoshop CS4.**
(DOC)

Protocol S2 **Grid drawing in tpdDig.**
(DOC)
