# Supplementary figures and images for: Phenotypic Divergence among West European Populations of Reed Bunting Emberiza schoeniclus: The Effects of Migratory and Foraging Behaviours
Source: PLoS One. 2013 May 7;8(5):e63248. doi: 10.1371/journal.pone.0063248 (PMC3646775; doi:10.1371/journal.pone.0063248)

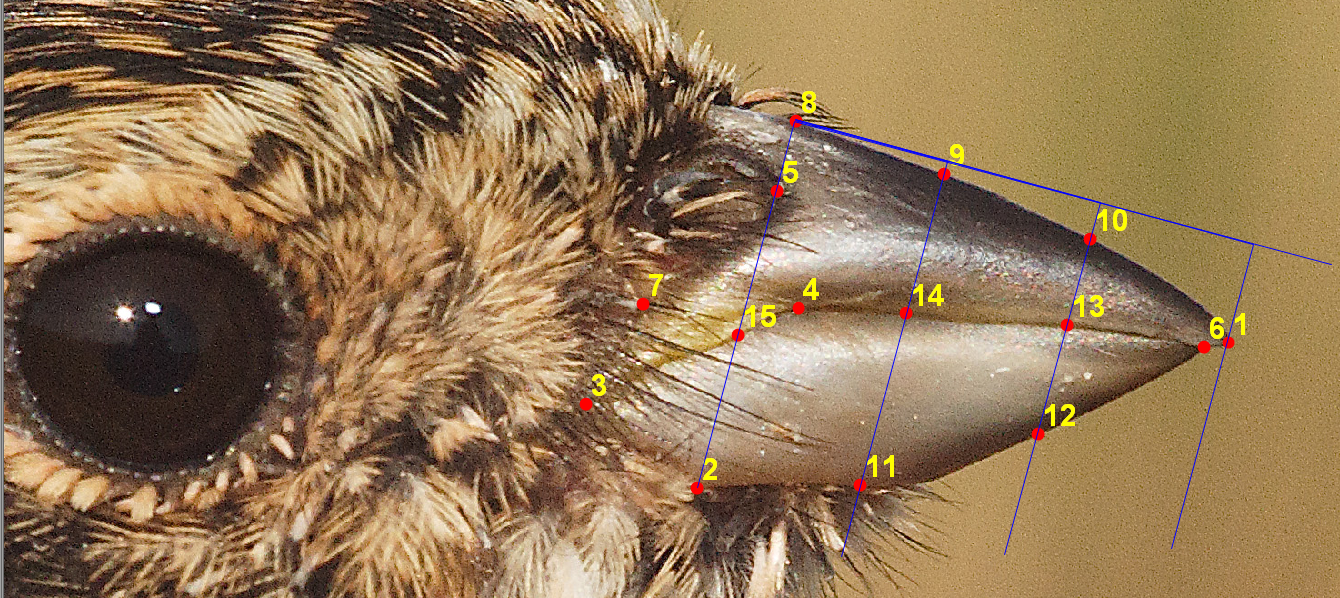

Supplement: Figure S2 — Location of the seven landmarks and eight semi-landmarks (calculated from the landmarks) used in geometric morphometric analyses. (TIF) [file pone.0063248.s002.tif]
