# Supplementary material for: Phenotypic Divergence among West European Populations of Reed Bunting Emberiza schoeniclus: The Effects of Migratory and Foraging Behaviours
Source: PLoS One. 2013 May 7;8(5):e63248. doi: 10.1371/journal.pone.0063248 (PMC3646775; doi:10.1371/journal.pone.0063248)
Supplement: Table S1 — Principal component analysis of bill size measurements, used to extract PCBILL. (DOC) [file pone.0063248.s003.doc]

| **Total Variance Explained** | | | | | | |
| --- | --- | --- | --- | --- | --- | --- |
| Component | Initial Eigenvalues | | | Extraction Sums of Squared Loadings | | |
|  | Total | % of Variance | Cumulative % | Total | % of Variance | Cumulative % |
| 1 | 1.802 | 60.071 | 60.071 | 1.802 | 60.071 | 60.071 |
| 2 | 0.994 | 33.142 | 93.213 |  |  |  |
| 3 | 0.204 | 6.787 | 100.000 |  |  |  |

| **Component Matrix** | |
| --- | --- |
|  | Component |
|  | 1 |
| Bill length | 0.231 |
| Bill depth | 0.947 |
| Bill width | 0.923 |
