# Supplementary material for: Phenotypic Divergence among West European Populations of Reed Bunting Emberiza schoeniclus: The Effects of Migratory and Foraging Behaviours
Source: PLoS One. 2013 May 7;8(5):e63248. doi: 10.1371/journal.pone.0063248 (PMC3646775; doi:10.1371/journal.pone.0063248)
Supplement: Table S2 — Principal component analysis of the isometrically-adjusted primary lengths, used to extract PC1WING and PC2WING, which represent wing convexity and wing pointedness, respectively. (DOC) [file pone.0063248.s004.doc]

| **Total Variance Explained** | | | | | | |
| --- | --- | --- | --- | --- | --- | --- |
| Component | Initial Eigenvalues | | | Extraction Sums of Squared Loadings | | |
|  | Total | % of Variance | Cumulative % | Total | % of Variance | Cumulative % |
| 1 | 4.194 | 46.597 | 46.597 | 4.194 | 46.597 | 46.597 |
| 2 | 1.885 | 20.948 | 67.545 | 1.885 | 20.948 | 67.545 |
| 3 | 1.058 | 11.759 | 79.304 |  |  |  |
| 4 | 0.607 | 6.745 | 86.049 |  |  |  |
| 5 | 0.415 | 4.614 | 90.663 |  |  |  |
| 6 | 0.316 | 3.512 | 94.175 |  |  |  |
| 7 | 0.263 | 2.921 | 97.096 |  |  |  |
| 8 | 0.143 | 1.585 | 98.681 |  |  |  |
| 9 | 0.119 | 1.319 | 100.000 |  |  |  |

| **Component Matrix** | | |
| --- | --- | --- |
|  | Component | |
|  | 1 | 2 |
| P9 | 0.297 | 0.675 |
| P8 | 0.433 | 0.777 |
| P7 | 0.576 | 0.564 |
| P6 | 0.595 | 0.308 |
| P5 | 0.698 | 0.005 |
| P4 | 0.831 | -0.240 |
| P3 | 0.846 | -0.351 |
| P2 | 0.853 | -0.314 |
| P1 | 0.782 | -0.365 |
