# Supplementary material for: Phenotypic Divergence among West European Populations of Reed Bunting Emberiza schoeniclus: The Effects of Migratory and Foraging Behaviours
Source: PLoS One. 2013 May 7;8(5):e63248. doi: 10.1371/journal.pone.0063248 (PMC3646775; doi:10.1371/journal.pone.0063248)
Supplement: Table S3 — Principal component analysis of body size measurements, used to extract PCSIZE. (DOC) [file pone.0063248.s005.doc]

| **Total Variance Explained** | | | | | | |
| --- | --- | --- | --- | --- | --- | --- |
| Component | Initial Eigenvalues | | | Extraction Sums of Squared Loadings | | |
|  | Total | % of Variance | Cumulative % | Total | % of Variance | Cumulative % |
| 1 | 2.067 | 51.679 | 51.679 | 2.067 | 51.679 | 51.679 |
| 2 | 0.988 | 24.691 | 76.370 |  |  |  |
| 3 | 0.716 | 17.894 | 94.264 |  |  |  |
| 4 | 0.229 | 5.736 | 100.000 |  |  |  |

| **Component Matrix** | |
| --- | --- |
|  | Component |
|  | 1 |
| Wing | 0.901 |
| Tail | 0.891 |
| Tarsus | 0.657 |
| Bill | 0.172 |
