# Supplementary material for: Phenotypic Divergence among West European Populations of Reed Bunting Emberiza schoeniclus: The Effects of Migratory and Foraging Behaviours
Source: PLoS One. 2013 May 7;8(5):e63248. doi: 10.1371/journal.pone.0063248 (PMC3646775; doi:10.1371/journal.pone.0063248)
Supplement: Table S4 — Descriptive statistics of morphological traits for each population, sex and age class. Individuals captured in Sweden did not differ from individuals of the nominate subspecies wintering in Portugal, and so they were lumped. (DOC) [file pone.0063248.s006.doc]

a) *E. s. schoeniclus*, adult males

|  | **N** | **Minimum** | **Maximum** | **Mean** | **Std. Error** |
| --- | --- | --- | --- | --- | --- |
| **Wing** | 20 | 78.00 | 87.00 | 83.2000 | 0.48177 |
| **P8** | 20 | 58.00 | 65.50 | 62.3500 | 0.40572 |
| **Tail** | 20 | 64.5 | 73.0 | 67.950 | 0.4321 |
| **Tarsus** | 20 | 18.5 | 20.5 | 19.540 | 0.1572 |
| **Bill length** | 20 | 12.5 | 14.2 | 13.310 | 0.0951 |
| **Weight** | 20 | 15.9 | 23.1 | 19.365 | 0.4590 |
| **Bill depth** | 19 | 4.9 | 5.8 | 5.326 | 0.0545 |
| **Bill width** | 19 | 4.2 | 5.4 | 4.632 | 0.0693 |
| **Bill shape** | 19 | 2.4 | 2.7 | 2.505 | 0.0223 |
| **Tail/Wing** | 20 | 0.7857 | 0.8598 | 0.816858 | 0.0041543 |
| **PC1_wing_** | 10 | -3.00935 | 0.39745 | -0.6806189 | 0.30247526 |
| **PC_bill_** | 19 | -1.00679 | 0.96456 | -0.1757131 | 0.11546877 |
| **PC_size_** | 20 | -0.08508 | 2.50380 | 1.1919476 | 0.14110635 |
| **RW1** | 12 | -0.08693661 | 0.02510300 | -0.0165500528 | 0.00972898694 |

b) *E. s. schoeniclus*, first-year males

|  | **N** | **Minimum** | **Maximum** | **Mean** | **Std. Error** |
| --- | --- | --- | --- | --- | --- |
| **Wing** | 18 | 80.00 | 87.00 | 82.3611 | 0.44245 |
| **P8** | 17 | 58.50 | 65.50 | 61.5000 | 0.40674 |
| **Tail** | 18 | 66.5 | 71.0 | 68.583 | 0.3688 |
| **Tarsus** | 18 | 18.5 | 21.1 | 19.967 | 0.1799 |
| **Bill length** | 18 | 12.4 | 13.9 | 13.156 | 0.1017 |
| **Weight** | 18 | 17.4 | 22.0 | 19.372 | 0.2723 |
| **Bill depth** | 18 | 4.8 | 5.5 | 5.206 | 0.0521 |
| **Bill width** | 18 | 3.9 | 5.5 | 4.700 | 0.1010 |
| **Bill shape** | 18 | 2.4 | 2.7 | 2.533 | 0.0181 |
| **Tail/Wing** | 18 | 0.8036 | 0.8650 | 0.832865 | 0.0038480 |
| **PC1_wing_** | 8 | -0.87762 | 0.24838 | -0.2442220 | 0.14420557 |
| **PC_bill_** | 18 | -1.36798 | 0.68003 | -0.2850498 | 0.14492142 |
| **PC_size_** | 18 | 0.69927 | 2.19804 | 1.3089171 | 0.09510728 |
| **RW1** | 13 | -0.06963698 | 0.02051702 | -0.0235799350 | 0.00762650852 |

c) *E. s. schoeniclus*, adult females

|  | **N** | **Minimum** | **Maximum** | **Mean** | **Std. Error** |
| --- | --- | --- | --- | --- | --- |
| **Wing** | 36 | 74.00 | 81.00 | 77.6250 | 0.28339 |
| **P8** | 33 | 53.50 | 61.00 | 57.6364 | 0.26046 |
| **Tail** | 36 | 59.0 | 69.0 | 64.694 | 0.3329 |
| **Tarsus** | 36 | 17.2 | 20.2 | 19.117 | 0.1204 |
| **Bill length** | 36 | 11.7 | 13.5 | 12.861 | 0.0786 |
| **Weight** | 36 | 15.6 | 19.5 | 17.078 | 0.1532 |
| **Bill depth** | 35 | 4.8 | 5.7 | 5.134 | 0.0357 |
| **Bill width** | 35 | 3.2 | 4.9 | 4.466 | 0.0528 |
| **Bill shape** | 35 | 2.3 | 2.8 | 2.503 | 0.0194 |
| **Tail/Wing** | 36 | 0.7815 | 0.8846 | 0.833442 | 0.0031959 |
| **PC1_wing_** | 16 | -1.65101 | 1.44060 | -0.2622198 | 0.19756004 |
| **PC_bill_** | 35 | -1.76389 | 0.38166 | -0.6208201 | 0.07793469 |
| **PC_size_** | 36 | -1.84005 | 1.06817 | -0.0950370 | 0.09467121 |
| **RW1** | 28 | -0.06126072 | 0.02208257 | -0.0232523349 | 0.00414189258 |

d) *E. s. schoeniclus*, first-year females

|  | **N** | **Minimum** | **Maximum** | **Mean** | **Std. Error** |
| --- | --- | --- | --- | --- | --- |
| **Wing** | 42 | 73.50 | 80.50 | 77.1190 | 0.22849 |
| **P8** | 40 | 53.00 | 60.50 | 57.1250 | 0.20782 |
| **Tail** | 41 | 60.0 | 67.5 | 64.354 | 0.2597 |
| **Tarsus** | 42 | 16.8 | 20.4 | 19.098 | 0.1193 |
| **Bill length** | 42 | 11.8 | 13.9 | 12.869 | 0.0756 |
| **Weight** | 39 | 13.7 | 22.2 | 17.156 | 0.2190 |
| **Bill depth** | 38 | 4.5 | 5.6 | 5.068 | 0.0413 |
| **Bill width** | 38 | 3.9 | 5.0 | 4.447 | 0.0444 |
| **Bill shape** | 38 | 2.3 | 2.9 | 2.539 | 0.0201 |
| **Tail/Wing** | 41 | 0.8000 | 0.8710 | 0.834758 | 0.0027068 |
| **PC1_wing_** | 13 | -1.67402 | 0.67211 | -0.4255270 | 0.18777186 |
| **PC_bill_** | 38 | -1.87810 | 0.27450 | -0.7054309 | 0.08560620 |
| **PC_size_** | 41 | -1.20273 | 0.92260 | -0.2120669 | 0.06896011 |
| **RW1** | 29 | -0.09397113 | 0.01772159 | -0.0321491578 | 0.00579737456 |

e) *E. s. lusitanica*, adult males

|  | **N** | **Minimum** | **Maximum** | **Mean** | **Std. Error** |
| --- | --- | --- | --- | --- | --- |
| **Wing** | 50 | 76.00 | 81.50 | 79.2800 | 0.20412 |
| **P8** | 43 | 55.50 | 61.00 | 58.9884 | 0.21789 |
| **Tail** | 47 | 61.0 | 69.5 | 65.436 | 0.2570 |
| **Tarsus** | 57 | 18.1 | 20.8 | 19.423 | 0.0743 |
| **Bill length** | 57 | 12.9 | 14.5 | 13.514 | 0.0445 |
| **Weight** | 58 | 15.5 | 19.4 | 17.886 | 0.1019 |
| **Bill depth** | 57 | 4.9 | 5.9 | 5.533 | 0.0294 |
| **Bill width** | 57 | 4.1 | 5.2 | 4.595 | 0.0345 |
| **Bill shape** | 56 | 2.3 | 2.7 | 2.441 | 0.0144 |
| **Tail/Wing** | 47 | 0.7673 | 0.8608 | 0.825018 | 0.0026897 |
| **PC1_wing_** | 10 | -1.09944 | 1.07280 | -0.1378834 | 0.20969688 |
| **PC_bill_** | 56 | -1.16143 | 0.89492 | 0.0677461 | 0.05885045 |
| **PC_size_** | 47 | -0.68210 | 1.55336 | 0.3982752 | 0.07001222 |
| **RW1** | 24 | -0.01552546 | 0.11164010 | 0.0413689387 | 0.00704651597 |

f) *E. s. lusitanica*, first-year males

|  | **N** | **Minimum** | **Maximum** | **Mean** | **Std. Error** |
| --- | --- | --- | --- | --- | --- |
| **Wing** | 21 | 73.00 | 79.00 | 75.8810 | 0.35001 |
| **P8** | 20 | 54.00 | 59.00 | 55.9500 | 0.28308 |
| **Tail** | 20 | 59.0 | 66.5 | 63.375 | 0.4244 |
| **Tarsus** | 21 | 18.4 | 20.5 | 19.310 | 0.1165 |
| **Bill length** | 21 | 12.5 | 14.6 | 13.310 | 0.1125 |
| **Weight** | 21 | 14.4 | 20.0 | 17.529 | 0.3147 |
| **Bill Depth** | 21 | 5.2 | 5.7 | 5.524 | 0.0284 |
| **Bill width** | 21 | 4.1 | 5.3 | 4.671 | 0.0614 |
| **Bill shape** | 21 | 2.2 | 2.8 | 2.419 | 0.0281 |
| **Tail/Wing** | 20 | 0.7922 | 0.8836 | 0.834273 | 0.0051950 |
| **PC1_wing_** | 3 | -2.04398 | 0.15733 | -0.6640759 | 0.69411514 |
| **PC_bill_** | 21 | -0.57204 | 0.51597 | 0.0728374 | 0.06462813 |
| **PC_size_** | 20 | -1.42868 | 0.64174 | -0.3291862 | 0.09985520 |
| **RW1** | 11 | -0.04037748 | 0.17203692 | 0.0546367634 | 0.01845410362 |

g) *E. s. lusitanica*, adult females

|  | **N** | **Minimum** | **Maximum** | **Mean** | **Std. Error** |
| --- | --- | --- | --- | --- | --- |
| **Wing** | 44 | 69.50 | 79.00 | 73.1932 | 0.27261 |
| **P8** | 37 | 51.00 | 59.00 | 54.2027 | 0.26242 |
| **Tail** | 42 | 57.5 | 68.0 | 62.060 | 0.3815 |
| **Tarsus** | 48 | 17.4 | 20.6 | 18.838 | 0.1022 |
| **Bill length** | 48 | 12.6 | 14.0 | 13.113 | 0.0506 |
| **Weight** | 49 | 13.9 | 19.1 | 15.904 | 0.1703 |
| **Bill depth** | 48 | 4.9 | 5.7 | 5.269 | 0.0273 |
| **Bill width** | 48 | 3.9 | 4.9 | 4.460 | 0.0351 |
| **Bill shape** | 48 | 2.3 | 2.7 | 2.488 | 0.0148 |
| **Tail/Wing** | 41 | 0.8014 | 0.9178 | 0.848270 | 0.0038054 |
| **PC1_wing_** | 8 | -0.84989 | 1.68372 | 0.5058748 | 0.30880820 |
| **PC_bill_** | 48 | -1.27930 | 0.43358 | -0.4200244 | 0.05455421 |
| **PC_size_** | 41 | -1.90889 | 0.67883 | -1.0202798 | 0.09048879 |
| **RW1** | 25 | -0.09368186 | 0.07977888 | -0.0061319140 | 0.00738256743 |

h) *E. s. lusitanica*, first-year females

|  | **N** | **Minimum** | **Maximum** | **Mean** | **Std. Error** |
| --- | --- | --- | --- | --- | --- |
| **Wing** | 21 | 68.00 | 73.50 | 70.6429 | 0.31837 |
| **P8** | 21 | 49.50 | 55.00 | 52.0238 | 0.32085 |
| **Tail** | 21 | 57.5 | 64.0 | 60.667 | 0.4116 |
| **Tarsus** | 21 | 17.9 | 19.3 | 18.519 | 0.0758 |
| **Bill length** | 21 | 12.5 | 13.4 | 13.148 | 0.0533 |
| **Weight** | 21 | 13.8 | 17.4 | 15.514 | 0.2190 |
| **Bill depth** | 21 | 4.5 | 5.6 | 5.214 | 0.0570 |
| **Bill width** | 20 | 4.2 | 4.9 | 4.530 | 0.0411 |
| **Bill shape** | 21 | 2.4 | 2.9 | 2.529 | 0.0260 |
| **Tail/Wing** | 21 | 0.8099 | 0.9197 | 0.858819 | 0.0047392 |
| **PC1_wing_** | 3 | -0.90078 | 1.05026 | -0.0528652 | 0.57749062 |
| **PC_bill_** | 20 | -1.05999 | 0.33917 | -0.3757133 | 0.08255773 |
| **PC_size_** | 21 | -2.29326 | -0.84524 | -1.6011798 | 0.09515144 |
| **RW1** | 12 | -0.05773664 | 0.03137872 | -0.0037080004 | 0.00856799138 |

i) *E. s. witherbyi*, adult males

|  | **N** | **Minimum** | **Maximum** | **Mean** | **Std. Error** |
| --- | --- | --- | --- | --- | --- |
| **Wing** | 8 | 75.00 | 81.00 | 79.0000 | 0.70711 |
| **P8** | 8 | 56.00 | 63.50 | 60.3750 | 0.91977 |
| **Tail** | 6 | 61.0 | 77.0 | 69.833 | 2.1042 |
| **Tarsus** | 9 | 17.4 | 21.3 | 20.089 | 0.3942 |
| **Bill length** | 7 | 12.1 | 13.5 | 12.900 | 0.1864 |
| **Weight** | 9 | 16.5 | 22.0 | 19.278 | 0.5570 |
| **Bill depth** | 8 | 6.2 | 6.7 | 6.488 | 0.0789 |
| **Bill width** | 7 | 5.0 | 7.2 | 5.829 | 0.3168 |
| **Bill shape** | 7 | 1.9 | 2.2 | 1.971 | 0.0474 |
| **Tail/Wing** | 6 | 0.8133 | 0.9872 | 0.883696 | 0.0232501 |
| **PC1_wing_** | 1 | 3.05784 | 3.05784 | 3.0578388 | 0.0 |
| **PC_bill_** | 7 | 1.32281 | 2.99059 | 2.0158336 | 0.25200454 |
| **PC_size_** | 5 | -0.59576 | 1.99815 | 1.1106233 | 0.44938200 |
| **RW1** | 1 | 0.18559707 | 0.18559707 | 0.1855970650 | 0.0 |

j) *E. s. witherbyi*, first-year males

|  | **N** | **Minimum** | **Maximum** | **Mean** | **Std. Error** |
| --- | --- | --- | --- | --- | --- |
| **Wing** | 11 | 74.00 | 84.00 | 79.6818 | 0.84819 |
| **P8** | 10 | 57.00 | 64.50 | 61.3500 | 0.71899 |
| **Tail** | 11 | 65.0 | 73.0 | 68.955 | 0.7150 |
| **Tarsus** | 12 | 19.8 | 21.3 | 20.617 | 0.1364 |
| **Bill length** | 12 | 12.1 | 15.3 | 13.300 | 0.2705 |
| **Weight** | 10 | 17.8 | 21.4 | 19.900 | 0.3445 |
| **Bill depth** | 12 | 6.1 | 6.9 | 6.517 | 0.0911 |
| **Bill width** | 12 | 4.7 | 6.8 | 5.925 | 0.2132 |
| **Bil shape** | 12 | 1.8 | 2.3 | 2.042 | 0.0452 |
| **Tail/Wing** | 11 | 0.8228 | 0.9189 | 0.865792 | 0.0082412 |
| **PC1_wing_** | 4 | -0.53504 | 3.57906 | 1.4094423 | 1.08514850 |
| **PC_bill_** | 12 | 0.78482 | 3.41628 | 2.1697482 | 0.22987731 |
| **PC_size_** | 11 | 0.14432 | 2.64153 | 1.3204985 | 0.24332499 |
| **RW1** | 5 | 0.06568295 | 0.17981081 | 0.1238896468 | 0.01809956408 |

k) *E. s. witherbyi*, adult females

|  | **N** | **Minimum** | **Maximum** | **Mean** | **Std. Error** |
| --- | --- | --- | --- | --- | --- |
| **Wing** | 6 | 71.50 | 75.00 | 73.4167 | 0.61124 |
| **P8** | 5 | 53.50 | 56.50 | 55.3000 | 0.51478 |
| **Tail** | 5 | 60.5 | 66.0 | 63.500 | 0.8944 |
| **Tarsus** | 9 | 18.8 | 20.3 | 19.367 | 0.1803 |
| **Bill legth** | 9 | 11.2 | 13.3 | 12.289 | 0.2003 |
| **Weight** | 6 | 15.3 | 19.0 | 17.183 | 0.6901 |
| **Bill depth** | 9 | 5.7 | 6.4 | 6.022 | 0.0722 |
| **Bill width** | 9 | 5.0 | 6.3 | 5.889 | 0.1522 |
| **Bill shape** | 9 | 1.9 | 2.3 | 2.056 | 0.0475 |
| **Tail/Wing** | 4 | 0.8533 | 0.8951 | 0.872863 | 0.0092379 |
| **PC1_wing_** | 1 | 0.67119 | 0.67119 | 0.6711887 | 0.0 |
| **PC_bill_** | 9 | 0.50526 | 1.97739 | 1.3773026 | 0.17538541 |
| **PC_size_** | 4 | -0.80161 | -0.14795 | -0.5610769 | 0.15151417 |
| **RW1** | 2 | 0.12229824 | 0.16834301 | 0.1453206255 | 0.02302238150 |

l) *E. s. witherbyi*, first-year females

|  | **N** | **Minimum** | **Maximum** | **Mean** | **Std. Error** |
| --- | --- | --- | --- | --- | --- |
| **Wing** | 5 | 72.00 | 75.00 | 74.0000 | 0.54772 |
| **P8** | 5 | 51.00 | 58.00 | 55.8000 | 1.25100 |
| **Tail** | 4 | 61.5 | 67.0 | 64.250 | 1.4506 |
| **Tarsus** | 6 | 18.7 | 20.8 | 20.083 | 0.3429 |
| **Bill length** | 5 | 11.7 | 13.2 | 12.420 | 0.2634 |
| **Weight** | 6 | 16.5 | 17.9 | 17.200 | 0.2380 |
| **Bill depth** | 5 | 5.7 | 6.5 | 6.080 | 0.1356 |
| **Bill width** | 5 | 5.0 | 6.7 | 5.780 | 0.3277 |
| **Bill shape** | 5 | 1.9 | 2.2 | 2.060 | 0.0510 |
| **Tail/Wing** | 4 | 0.8200 | 0.9054 | 0.862680 | 0.0227994 |
| **PC1_wing_** | 0 |  |  |  |  |
| **PC_bill_** | 5 | 0.61059 | 2.42598 | 1.3810382 | 0.31861776 |
| **PC_size_** | 4 | -0.80487 | 0.12202 | -0.3493920 | 0.19143066 |
| **RW1** | 0 |  |  |  |  |

m) *E. s. schoeniclus*, adult males from the U. K.

|  | **N** | **Minimum** | **Maximum** | **Mean** | **Std. Error** |
| --- | --- | --- | --- | --- | --- |
| **Wing** | 10 | 79.50 | 85.00 | 82.2500 | 0.60208 |
| **P8** | 10 | 59.50 | 65.50 | 62.3000 | 0.55377 |
| **Tail** | 10 | 65.0 | 71.0 | 68.000 | 0.5323 |
| **Tarsus** | 10 | 18.9 | 24.5 | 20.760 | 0.5860 |
| **Bill length** | 8 | 12.1 | 13.3 | 12.688 | 0.1481 |
| **Weight** | 10 | 18.4 | 22.0 | 19.490 | 0.3446 |
| **Bill depth** | 9 | 4.9 | 5.5 | 5.156 | 0.0580 |
| **Bill width** | 6 | 3.8 | 4.4 | 4.083 | 0.0910 |
| **Bill shape** | 7 | 2.3 | 2.6 | 2.500 | 0.0378 |
| **Tail/Wing** | 10 | 0.8095 | 0.8625 | 0.826845 | 0.0048451 |
| **PC1_wing_** | 8 | -0.00831 | 1.26194 | 0.4743480 | 0.19978963 |
| **PC_bill_** | 5 | -1.18673 | -0.93583 | -1.0518969 | 0.04897743 |
| **PC_size_** | 8 | 0.22593 | 2.12748 | 1.3126658 | 0.19502805 |
| **RW1** | 9 | -0.08583785 | 0.03274251 | -0.0127423861 | 0.01185048262 |

n) *E. s. schoeniclus*, first-year males from the U. K.

|  | **N** | **Minimum** | **Maximum** | **Mean** | **Std. Error** |
| --- | --- | --- | --- | --- | --- |
| **Wing** | 20 | 79.00 | 85.00 | 81.1000 | 0.34527 |
| **P8** | 20 | 59.00 | 65.00 | 61.1000 | 0.32967 |
| **Tail** | 20 | 62.0 | 69.0 | 65.725 | 0.4537 |
| **Tarsus** | 20 | 19.3 | 23.3 | 20.495 | 0.2864 |
| **Bill length** | 16 | 11.8 | 13.1 | 12.638 | 0.1060 |
| **Weight** | 20 | 18.0 | 21.8 | 19.470 | 0.2394 |
| **Bill depth** | 20 | 4.5 | 5.4 | 4.905 | 0.0559 |
| **Bill width** | 15 | 3.5 | 4.6 | 4.193 | 0.0836 |
| **Bill shape** | 16 | 2.4 | 2.9 | 2.600 | 0.0387 |
| **Tail/Wing** | 20 | 0.7590 | 0.8500 | 0.810612 | 0.0059847 |
| **PC1_wing_** | 18 | -2.04722 | 1.61758 | -0.0602602 | 0.22212294 |
| **PC_bill_** | 13 | -1.71273 | -0.42887 | -1.1671276 | 0.12731486 |
| **PC_size_** | 16 | 0.23691 | 1.61369 | 0.8638221 | 0.11584239 |
| **RW1** | 16 | -0.10391997 | 0.00005960 | -0.0270659251 | 0.00606408108 |

o) *E. s. schoeniclus*, adult females from the U. K.

|  | **N** | **Minimum** | **Maximum** | **Mean** | **Std. Error** |
| --- | --- | --- | --- | --- | --- |
| **Wing** | 2 | 74.00 | 78.00 | 76.0000 | 2.00000 |
| **P8** | 2 | 54.50 | 58.50 | 56.5000 | 2.00000 |
| **Tail** | 2 | 60.0 | 66.0 | 63.000 | 3.0000 |
| **Tarsus** | 2 | 19.5 | 20.2 | 19.850 | 0.3500 |
| **Bill length** | 0 |  |  |  |  |
| **Weight** | 2 | 16.0 | 17.8 | 16.900 | 0.9000 |
| **Bill depth** | 2 | 4.4 | 5.0 | 4.700 | 0.3000 |
| **Bill width** | 2 | 3.9 | 4.5 | 4.200 | 0.3000 |
| **Bill shape** | 0 |  |  |  |  |
| **Tail/Wing** | 2 | 0.8108 | 0.8462 | 0.828482 | 0.0176715 |
| **PC1_wing_** | 2 | 0.52028 | 0.54866 | 0.5344698 | 0.01418934 |
| **PC_bill_** | 0 |  |  |  |  |
| **PC_size_** | 0 |  |  |  |  |
| **RW1** | 2 | -0.06999003 | -0.06065344 | -0.0653217345 | 0.00466829950 |

p) *E. s. schoeniclus*, first-year females from the U. K.

|  | **N** | **Minimum** | **Maximum** | **Mean** | **Std. Error** |
| --- | --- | --- | --- | --- | --- |
| **Wing** | 10 | 73.00 | 79.00 | 75.7000 | 0.54365 |
| **P8** | 10 | 52.00 | 59.00 | 56.4500 | 0.63004 |
| **Tail** | 10 | 58.0 | 65.0 | 62.600 | 0.7446 |
| **Tarsus** | 10 | 18.1 | 22.2 | 20.020 | 0.4912 |
| **Bill length** | 6 | 11.8 | 13.4 | 12.467 | 0.2124 |
| **Weight** | 10 | 15.6 | 19.2 | 17.390 | 0.3391 |
| **Bill depth** | 10 | 4.1 | 5.2 | 4.790 | 0.1130 |
| **Bill width** | 7 | 4.0 | 4.3 | 4.186 | 0.0404 |
| **Bill shape** | 6 | 2.4 | 2.7 | 2.533 | 0.0422 |
| **Tail/Wing** | 10 | 0.7945 | 0.8553 | 0.826838 | 0.0065494 |
| **PC1_wing_** | 7 | -0.79604 | 1.75062 | 0.4512214 | 0.39547803 |
| **PC_bill_** | 4 | -1.27801 | -0.88836 | -1.1287710 | 0.09184407 |
| **PC_size_** | 6 | -0.70443 | 0.91425 | -0.1466503 | 0.24567195 |
| **RW1** | 9 | -0.09061147 | 0.02586803 | -0.0343655421 | 0.01183131005 |
