# Supplementary material for: Phenotypic Divergence among West European Populations of Reed Bunting Emberiza schoeniclus: The Effects of Migratory and Foraging Behaviours
Source: PLoS One. 2013 May 7;8(5):e63248. doi: 10.1371/journal.pone.0063248 (PMC3646775; doi:10.1371/journal.pone.0063248)
Supplement: Protocol S1 — Photograph editing in Photoshop CS4. (DOC) [file pone.0063248.s007.doc]

1. When necessary, the photograph was flipped horizontally so that all bills would face right;
2. Zoom Level was set at 100%;
3. When necessary, the image was rotated so that pupil center and bill tip would lay exactly on the same imaginary horizontal line;
4. The picture was cropped so that the output would have a fixed width to height ratio of 5/3, the same distance left of the bill gape and right of the bill tip, and the same distance above the culmen and below the gonys (to make the best use of tpsDig window shape);
5. Each photograph was saved in jpg format using the ring number of the depicted bird as file name.
