# Supplementary material for: Phenotypic Divergence among West European Populations of Reed Bunting Emberiza schoeniclus: The Effects of Migratory and Foraging Behaviours
Source: PLoS One. 2013 May 7;8(5):e63248. doi: 10.1371/journal.pone.0063248 (PMC3646775; doi:10.1371/journal.pone.0063248)
Supplement: Protocol S2 — Grid drawing in tpdDig. (DOC) [file pone.0063248.s008.doc]

1. Click the “Make angle measurements” button and next the “Draw background curves” button. Starting on landmark 2 draw a straight line going through landmark 5 and ending in the culmen ridge and then another straight line at a 90 degree angle from the first extending beyond bill tip;
2. Click the “Make linear measurements button” and measure the minimum distance between landmark 1 and the line going from landmark 2 to landmark 5 and to the culmen and divide the value by three;
3. Measure this last value from the 90 degree angle along the line going towards bill tip;
4. Use the “Make angle measurements” and the “Draw background curves” buttons again to draw a new straight line from the 90 degree angle to the measured point (over part of the preexisting line) and then another straight line at a 90 degree angle from the first extending down beyond the gonys;
5. Repeat the above procedure three times (the last one is for accuracy checking only);
6. Digitize semi-landmarks 8 to 15 in the order illustrated in Figure S2.
